# Genome-Wide Small RNA Analysis of Soybean Reveals Auxin-Responsive microRNAs that are Differentially Expressed in Response to Salt Stress in Root Apex

**DOI:** 10.3389/fpls.2015.01273

**Published:** 2016-01-18

**Authors:** Zhengxi Sun, Youning Wang, Fupeng Mou, Yinping Tian, Liang Chen, Senlei Zhang, Qiong Jiang, Xia Li

**Affiliations:** ^1^State Key Laboratory of Plant Cell and Chromosome Engineering, Center for Agricultural Resources Research, Institute of Genetics and Developmental Biology, Chinese Academy of SciencesShijiazhuang, China; ^2^University of Chinese Academy of SciencesBeijing, China; ^3^State Key Laboratory of Agricultural Microbiology, College of Plant Science and Technology, Huazhong Agricultural UniversityWuhan, China

**Keywords:** *Glycine max*, root meristem, microRNA (miRNA), salt stress, auxin

## Abstract

Root growth and the architecture of the root system in *Arabidopsis* are largely determined by root meristematic activity. Legume roots show strong developmental plasticity in response to both abiotic and biotic stimuli, including symbiotic rhizobia. However, a global analysis of gene regulation in the root meristem of soybean plants is lacking. In this study, we performed a global analysis of the small RNA transcriptome of root tips from soybean seedlings grown under normal and salt stress conditions. In total, 71 miRNA candidates, including known and novel variants of 59 miRNA families, were identified. We found 66 salt-responsive miRNAs in the soybean root meristem; among them, 22 are novel miRNAs. Interestingly, we found auxin-responsive *cis*-elements in the promoters of many salt-responsive miRNAs, implying that these miRNAs may be regulated by auxin and auxin signaling plays a key role in regulating the plasticity of the miRNAome and root development in soybean. A functional analysis of miR399, a salt-responsive miRNA in the root meristem, indicates the crucial role of this miRNA in modulating soybean root developmental plasticity. Our data provide novel insight into the miRNAome-mediated regulatory mechanism in soybean root growth under salt stress.

## Introduction

The root is an important organ that not only supports the aerial parts of plants, but also absorbs water and nutrients for plant growth and survival. The growth rate and architecture of roots are dynamically regulated by internal and external cues, and they are largely determined by the activity of the root apical meristem (RAM) (De Tullio et al., 2010; Ubeda-Tomas and Bennett, 2010; Petricka et al., 2012). The RAM is localized at the root tip and harbors a stem cell niche that serves as the source of new cells for continuous apical root growth involving iterative processes of cell division, elongation, and differentiation (Dinneny and Benfey, 2008; Perilli et al., 2012). Maintenance of the optimal size and activity of the RAM is crucial for proper apical root growth and root system architecture.

The fate of root meristem cells is determined by both developmental and environmental cues in *Arabidopsis* plants. Among the developmental signals, auxin is a central regulator that balances the rate of cell division and differentiation through interplay with other plant growth regulators, especially cytokinin (Kerk et al., 2000; Aloni et al., 2006; Zhang et al., 2013). In recent decades, an auxin-mediated regulatory pathway has been identified; in this pathway, auxin regulates the establishment of the stem cell niche and activity of the RAM through PLETHORA1/2, an AP2/EREBP family transcription factor (Ding and Friml, 2010). Notably, cell fate and the subsequent activity of the RAM are also controlled by extrinsic signals, including abiotic stresses (West et al., 2004; Ji et al., 2014). Under conditions of salt stress, the root is the first plant organ to encounter the stress and sense the salt signal (Liu et al., 2015). It is well known that root growth is greatly inhibited by salt in various plant species and that root growth inhibition is mainly due to reduced activity of the RAM (Burgos et al., 2004; Fernández-Marcos et al., 2011; Liu et al., 2013). Recently, it was shown that the salt-induced reduction in RAM activity is mediated by auxin and auxin signaling in *Arabidopsis* (Liu et al., 2015). However, the molecular mechanism through which RAM activity is modulated in response to salt stress remains largely unknown.

microRNAs (miRNAs) are small non-coding RNAs that modulate various biological processes at the post-transcriptional level by mediating mRNA degradation or translational repression (Bartel, 2004). Recent global analyses of miRNA expression profiles have demonstrated that miRNAs play a key role in modulating plant root plastic development in response to biotic and abiotic stimuli (Ding et al., 2009; Lelandais-Briere et al., 2009; Lu et al., 2014). Many miRNAs, including miR156, miR172, and miR390, which are responsive to salt stress, have been identified in various plants (Khraiwesh et al., 2012; Sunkar et al., 2012; Cui et al., 2014; Xie et al., 2014; Li et al., 2016). Recent studies have revealed that miRNAs not only regulate RAM activity under normal conditions, but also mediate the RAM response to salt stress in *Arabidopsis* and *Medicago truncatula* (Bazin et al., 2013; Bustos-Sanmamed et al., 2013; Singh et al., 2014). These results highlight the central role of miRNAs and the miRNA-mediated regulatory network in the programming of RAM activity and root developmental plasticity in response to salt stress. Owing to the pivotal importance of miRNAs in RAM activity and root plastic development in response to salt stress, elucidation of the miRNA-mediated regulatory network is of great importance.

Soybean is a major economic crop worldwide; however, the yield is greatly affected by soil salinity (Yasuta and Kokubun, 2014). Previous studies have shown that soybean copes with salt stress using physiological mechanisms that are similar to those in *Arabidopsis*, including the maintenance of ion homeostasis, adjustment of cellular responses to osmotic stress, and metabolic responses (Im et al., 2012; Guan et al., 2014; Rao et al., 2014). Soybean is a legume that possesses the ability to form root nodules that can fix N_2_ from the atmosphere through a symbiotic interaction with rhizobia (Ferguson et al., 2010). Therefore, studies of root growth and development in response to salt stress are of the utmost significance in soybean genetics. Despite great effort to identify the relevant genes and/or proteins using various approaches (Aghaei et al., 2009; Ge et al., 2010; Sobhanian et al., 2010), only a few genes, including that encoding respiratory alternative oxidase and *GmSALT3*, have been shown to mediate root responses to salt and salt tolerance in soybean (Guan et al., 2014). Knowledge of the gene regulatory network underlying the response of the RAM in soybean to salt, especially the environmental plasticity of the miRNAome, is lacking.

Here, we present the results of a global analysis of deep sequencing data from two miRNA libraries prepared from soybean root apexes treated with or without salt stress. Genome-wide identification of the miRNAs revealed that they are involved in RAM regulation and that the RAM miRNAome with salt stress shows remarkable plasticity compared to RAM miRNAome without salt stress. In addition to conserved miRNAs associated with the root response to salt stress in plants, we also found several miRNAs that may specifically mediate RAM reprogramming in soybean. Furthermore, our results reveal an important role for the auxin-miRNA regulatory network in the RAM response to salt stress. We also identified a novel role for miR399 in root developmental plasticity under salt stress. Our results highlight the vital role of the miRNA-mediated regulatory network in RAM reprogramming and root adaptations to saline soil.

## Materials and methods

### Plant materials and treatment methods

Seeds (*Glycine max* [L.] Merrill cv. Williams 82) were surface-sterilized with 75% ethanol for 1 min, followed by chlorine for 8 h. The sterilized seeds were germinated in plastic pots filled with 400 ml of solid B5 medium containing 0 or 75 mM NaCl. Seedlings were grown in a greenhouse under 16 h of light at 28°C for 5 days. Root tips (0.5 cm) were collected from the plants for high-throughput sequencing, histological sectioning, and indoleacetic acid (IAA) measurement. For 2,4-D treatment, five-day-old seedlings were transferred to liquid B5 medium supplemented with or without 1 μM 2,4-D. After 3 days, root tips were collected.

### Measurement of IAA

To determine the IAA content, root tips (0.5 cm) were collected from the medium containing 0 or 75 mM NaCl. IAA was extracted and measured as described by Fu et al. (2012) with modifications. After extraction and purification, the samples were analyzed by liquid chromatography-tandem mass spectrometry using a system that included an Acquity Ultra Performance Liquid Chromatograph (Waters Corp., Milford, MA) and a triple quadrupole tandem mass spectrometer (Quattro Premier XE; Waters Corp.).

### Longitudinal sectioning of the roots

To prepare longitudinal sections of the roots, root tips were collected from the medium with or without 75 mM NaCl and then fixed in formalin/acetic acid/alcohol. The root segments were dehydrated at room temperature in a gradient series of ethanol solutions, followed by two changes of Clear-Rite 3 (Sigma-Aldrich Corp., St. Louis, MO) for 1 h each, and finally embedded in Paraplast (Leica Biosystems Nussloch Gmbh, Nußloch, Germany) at 62°C. Sectioning was performed using a microtome. Sections were cut every 20 μm and then stained with 0.1% (w/v) Eosin Y. The sections were observed under an Olympus CX31 biological microscope (Tokyo, Japan).

### Small RNA library construction and high-throughput sequencing

For small RNA library construction, root tips (0.5 cm) were harvested from B5 medium supplemented with (SR) or without (CR) 75 mM NaCl, and immediately frozen in liquid N_2_. The samples collected from the two libraries were sent to Biomarker Technologies Co. (Beijing) for analysis. Small RNAs were isolated from stressed (SR) and non-stressed (CR) root tips using a TruSeq Small RNA Sample Prep Kit (Illumina Inc., San Diego, CA) to construct the libraries. The sequence data were analyzed using miRDeep2 by Biomarker Technologies Co. according to the manufacturer's protocols. The read abundances of miRNAs in the two libraries were normalized to the reads per kilobase of exon model per million mapped reads value (normalized expression = actual miRNA count/total count of clean reads × normalized one order of magnitude). The *P*-value threshold was determined by the false discovery rate (FDR) to account for multiple tests of significance. To determine the functional annotation of the genes targeted by the differentially expressed miRNAs, a BLAST alignment was performed by searching the NR, SwissProt, GO, COG, and KEGG protein databases. This experiment consisted of three independent biological replicates.

### Real-time quantitative polymerase chain reaction (qRT-PCR) analysis

Total RNA was extracted from root tips using Trizol reagent (Tiangen Biotech [Beijing] Co., Ltd., Beijing, China), and then treated with DNase I to remove contaminating genomic DNA. Stem-loop-specific reverse transcription for miRNAs was performed as described previously (Chen et al., 2005; Kulcheski et al., 2010). First-strand cDNA was synthesized from the total RNA using a FastQuant RT Kit (TransGen Biotech Inc., Beijing, China). qRT-PCR assays were performed using SuperReal PreMix Plus (SYBR Green; Tiangen Biotech [Beijing] Co., Ltd.). Soybean miR1515a was used as a reference miRNA to normalize the samples as described (Turner et al., 2013). The primers used for cDNA synthesis and qRT-PCR are listed in Table S5 online.

### Vector construction and soybean hairy root transformation

For the gma-miR399a overexpression construct, the pre-miRNA fragment (94 base pairs [bp]) of gma-miR399a was amplified and inserted into the Gateway® binary vector pGWB2. The primers used for plasmid construction are listed in Table S5 Soybean transformation to generate hairy root composite plants was done according to a previously described method with modifications (Kereszt et al., 2007; Jian et al., 2009). After co-cultivation with *Agrobacteriom rhizogenes* K599 containing constructed vector, the explants were transferred to MS/2 medium supplemented with or without 75 mM NaCl. Ten days after transplantation, the expression of miR399a was analyzed in hairy roots harboring the 35::miR399a construct using qRT-PCR. The primary root length and number of lateral roots per positive hairy root were estimated.

### Bioinformatics analysis

To analyze the *cis*-elements of the miRNAs, 2000 bp of sequence located upstream of each salt stress-responsive miRNA was chosen as a putative promoter sequence for analysis using PLACE (http://www.dna.affrc.go.jp/PLACE).

### Statistical analysis

All data were analyzed using SigmaPlot 10.0 (Systat Software, Inc., Chicago, IL) and GraphPad Prism 5 (GraphPad Software, Inc., La Jolla, CA) software. Student's *t*-test was performed using GraphPad Prism 5.

## Results

### Salt stress suppresses the activity of the RAM and increases IAA in the RAM

**Figure 1:**
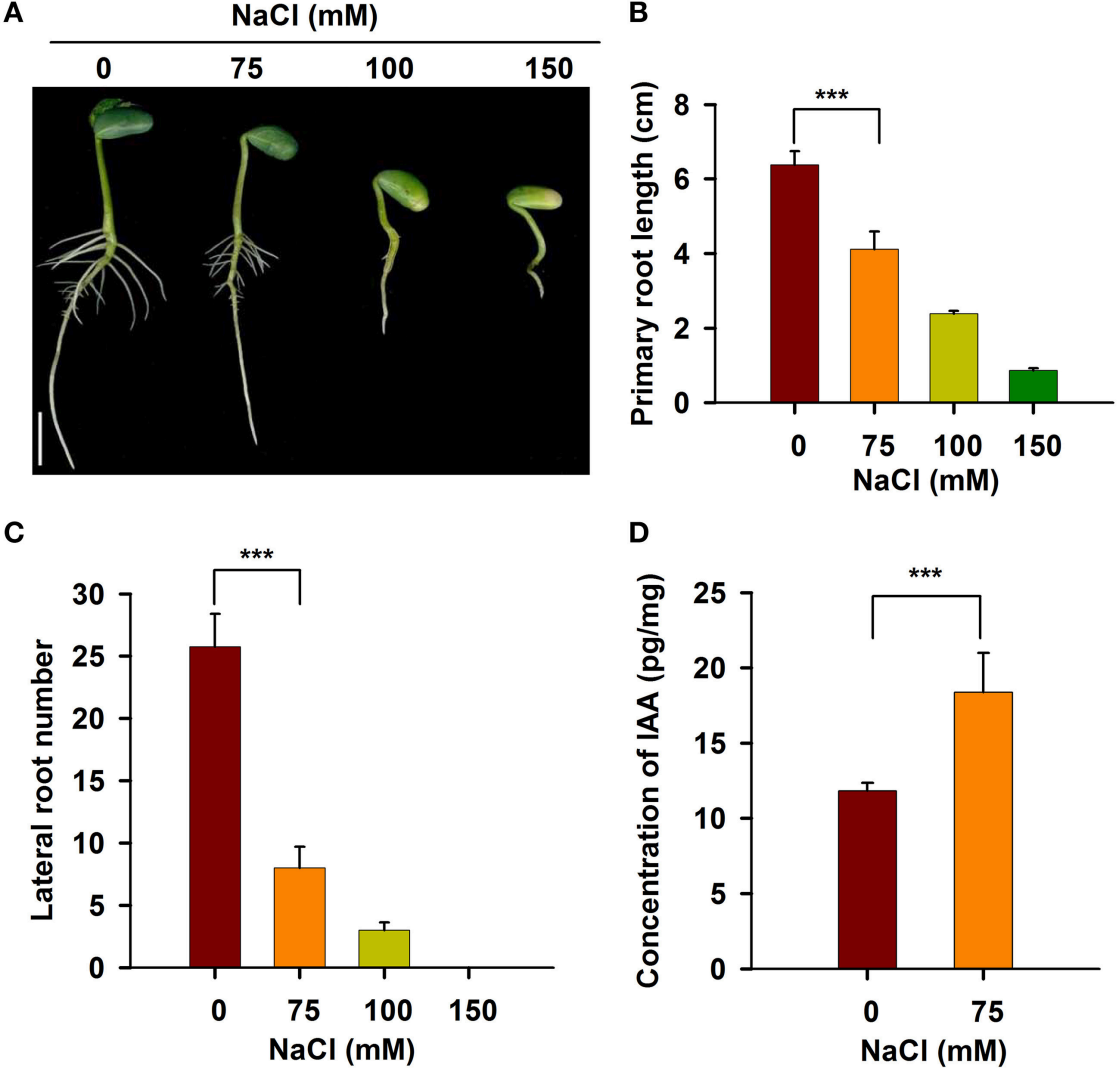
**The development of soybean root was affected under salt stress**. Five-day-old seedlings germinated on B5 medium containing different concentrations of NaCl were taken photos **(A)**. Bar = 1 cm. The length of primary root **(B)** and the number of lateral root **(C)** were counted. **(D)** IAA content was measured using LC/MS-MS method. Error bars indicate the standard deviation. Statistically significant differences (Student's *t*-test) are indicated as follows: “^***^” (*P* < 0.001).

To investigate the responses of roots to salt stress, we performed a systematic study of soybean root development. As shown in Figure 1A, the roots of young soybean seedlings were sensitive to salt stress. When treated with 75 mM NaCl, both primary root growth and lateral root development were significantly inhibited. When the NaCl concentration was increased to 100 mM, the length of the primary root in the seedlings was reduced by about 70% (Figures 1A,B), and the number of lateral roots was significantly decreased compared with the control (Figures 1A,C). When the concentration of NaCl was increased to 150 mM, primary and lateral roots were almost completely and completely inhibited, respectively (Figures 1A–C). These results indicate that a NaCl concentration of 100 mM or greater imposed severe stress on the soybean seedlings. Therefore, 75 mM NaCl was used in all subsequent studies of root plastic development.

It is well known that the activity of the RAM determines root growth (Grunewald et al., 2012; Perilli et al., 2012; Petricka et al., 2012; D'alessandro et al., 2015). To investigate whether the growth inhibition of soybean roots by salt is due to a reduction in root meristem activity, we measured the length of the meristem zone in longitudinally cut root tips from seedlings grown in 0 or 75 mM NaCl. The length of the meristem in the control roots was approximately 0.5 cm, whereas the meristems of the stressed roots were significantly shorter (Figure S1). These data suggest that the salt stress-induced growth retardation of roots and developmental plasticity of the root system are mainly caused by the suppression of RAM activity.

IAA plays a key role in the maintenance of root meristem activity (Jiang and Feldman, 2002; Marchant et al., 2002; Lavenus et al., 2013). To elucidate the physiological mechanism underlying salt-induced growth retardation, we measured the IAA content in the RAM of non-stressed and stressed soybean seedlings. As shown in Figure 1D, upon salt stress, the content of IAA in the RAM region was significantly increased. These results indicate that the reduced activity of the RAM and root growth in response to salt stress are related to the increased IAA content in the RAM of stressed seedlings.

### High-throughput sequencing and annotation of miRNAs in root apexs under salt stress

To explore the molecular mechanism through which soybean RAM activity is modulated in response to salt stress, we performed a genome-wide analysis of the miRNAs in the soybean RAM under normal and salt stress conditions. Two libraries constructed from the total RNA of roots treated with and without 75 mM NaCl were subjected to Illumina Solexa deep sequencing. Approximately 19,895,358 and 20,028,331 total reads were generated from the control and salt-stressed RAM libraries, respectively (Table 1). Among the reads, those with sequences containing fewer than 18 nucleotides (nt) (1,371,822 and 1,834,851 for the control and stressed RAM libraries, respectively) or more than 30 nt (2,967,654 and 3,539,771 for the control and stressed RAM libraries, respectively) were not considered. Adaptor sequences, low-quality tags, and contaminants were also removed, resulting in 15,553,439 and 14,653,373 total clean reads for the control and stressed RAM libraries, respectively. These clean reads were perfectly matched to the soybean genome and accounted for approximately 78.176 and 73.163% of the total reads in the control and stressed RAM libraries, respectively (Table 1).

**Table 1:** **Statistic of sRNA in CR and SR sequencing libraries**.

**Type**	**CR**		**SR**	
	**Count**	**Percentage**	**Count**	**Percentage**
Total reads	19,895,358		20,028,331	
Low quality	0	0	0	0
“N” reads	2443	0.012%	336	0.002%
Length <18	1,371,822	6.895%	1,834,851	9.161%
Length >30	2,967,654	14.916%	3,539,771	17.674%
Clean reads	15,553,439	78.176%	14,653,373	73.163%

**Figure 2:**
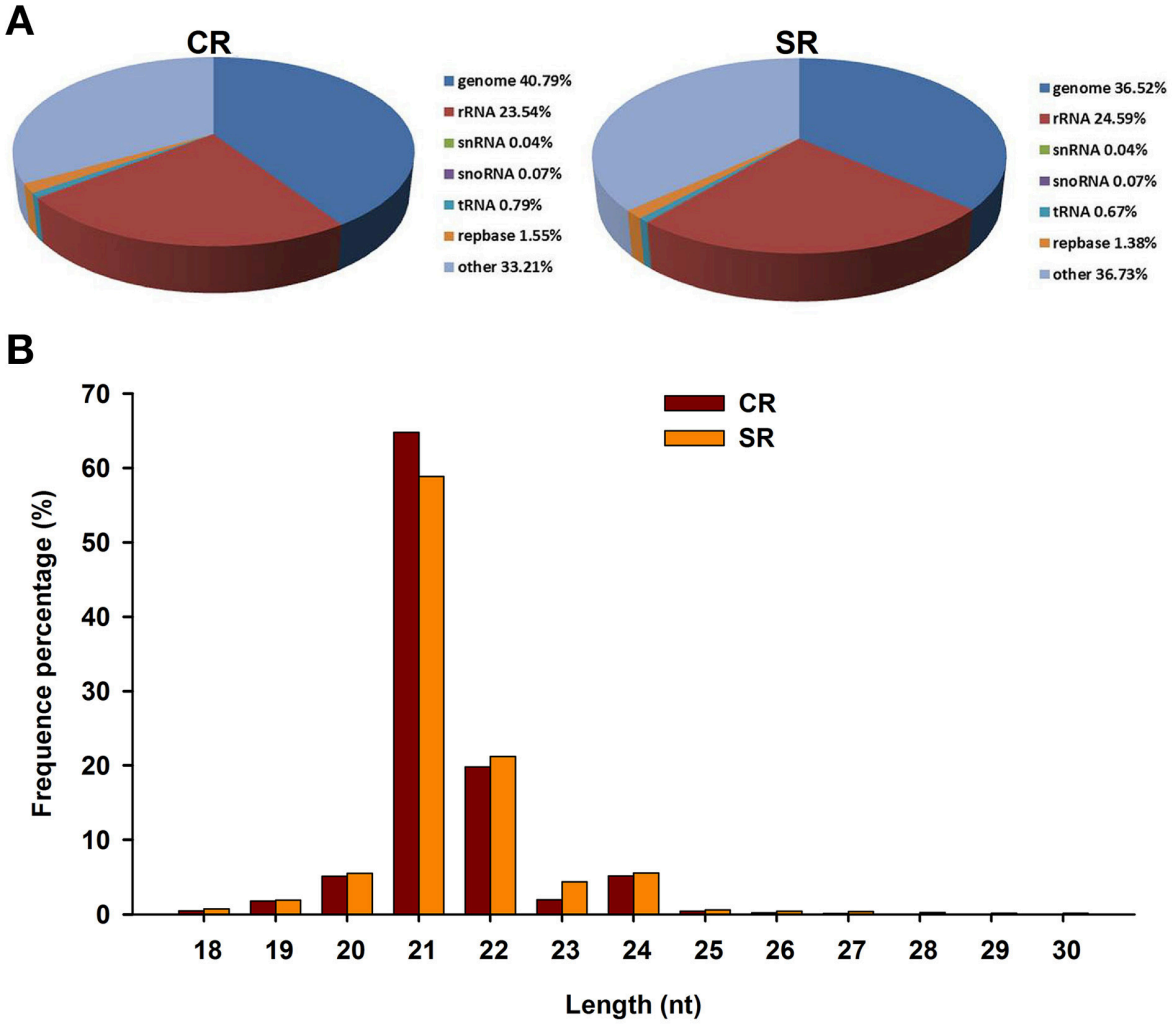
**Characteristics of small RNAs in CR and SR libraries through Solexa sequencing in CR**. **(A)** Distribution of small RNA reads among different RNA categories in CR and SR libraries. Genome group contained extron, intron, and miRNA. **(B)** Size distribution of small RNAs in CR and SR libraries.

By alignment with the GenBank and Rfam databases, the clean small RNAs were classified into five different categories (Figure 2A). Based on the numbers of unique reads, the ranking of the RNA types, in descending order, was: rRNAs, tRNAs, snoRNAs, and snRNAs. This pattern was observed in both libraries, although the exact numbers in each category were different. In comparing the miRNAs between the two libraries, we found that the size distribution of the miRNAs in the stressed RAM library was comparable to that in the control RAM library. More than 70% of the genome-mapped miRNAs were between 18 and 30 nt in length; however, the majority of the miRNAs in both libraries were 21–22 nt in length (Figure 2B). Notably, the total number of miRNAs produced from the stressed RAM library was 4.27% less than that produced from the control library (Figure 2A). In accordance with this, the miRNAs that showed the greatest reduction due to salt stress were 21 nt in length, suggesting a role for these miRNAs in the RAM response to salt stress in soybean.

### Identification and validation of known salt-responsive miRNAs

**Figure 3:**
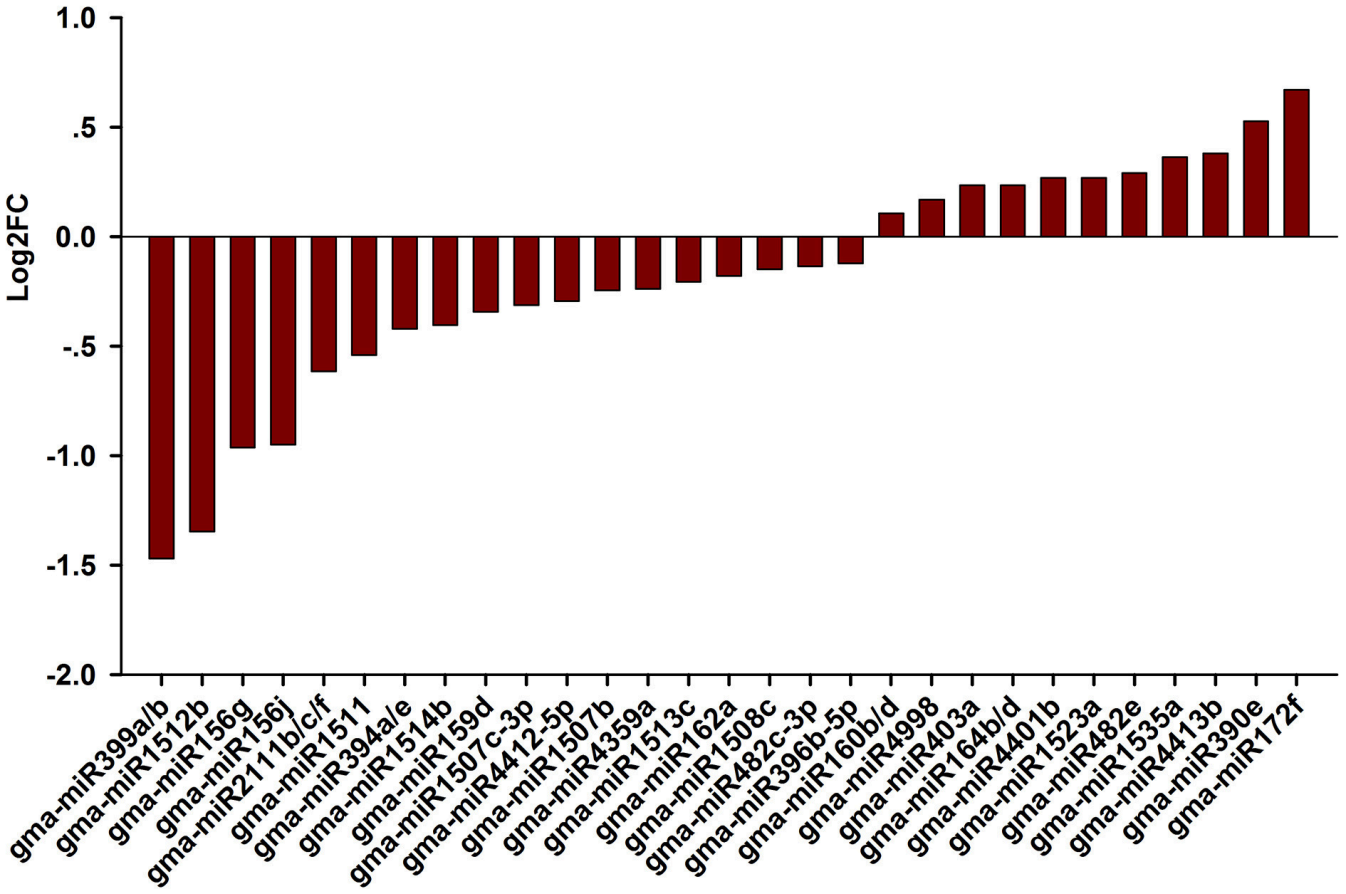
**Fold change of known miRNAs expression analysis in CR and SR libraries**. Logarithmic fold change of known miRNAs expression in response to salt stress according the sequencing results from CR and SR libraries.

Next, we attempted to identify salt stress-responsive miRNAs. First, we aligned the clean reads with a plant miRNA database using miRDeep2 to pick up known miRNAs. Overall, 46 known miRNAs were found and 41 known miRNAs were shared between the libraries (Table S1). Amongst them, abundance of gma-miR396b-5p, gma-miR319l, and gma-miR1511 wasvery high in the two libraries, suggesting an essential role for these miRNAs in the RAM of soybean. Salt-responsive miRNAs were then identified based on two criteria: a log2 fold change (log2FC) >0.1 or < −0.1 and an FDR < 0.01. Ultimately, 36 known miRNAs belonging to different conserved families that were responsive to salt stress in three biological replicates were identified (Figure 3; Table S1). Among them, 14 known miRNAs were up-regulated by salt stress, while 22 miRNAs were down-regulated by salt stress; in addition, 4 and 1 known miRNAs were specifically expressed in roots treated without or with salt stress, respectively (Table S1). When the selection criteria became more stringent (log2FC >0.5 or < −0.5), nine salt stress-responsive miRNAs in the RAM region were obtained. Among them, the most significantly up-regulated miRNAs were miR172f, followed by miR390e. In contrast, miR399a/b was the most repressed miRNA under salt stress, followed by miR1512b, miR156g, and miR156j (Figure 3).

**Figure 4:**
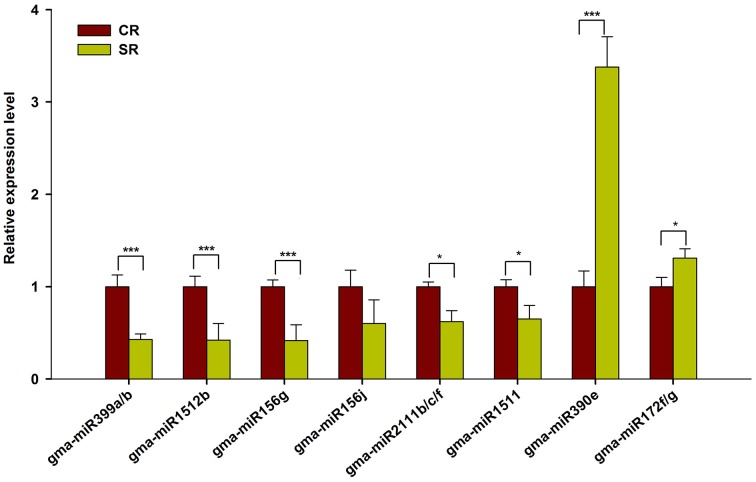
**qRT-PCR analysis of known miRNAs in response to salt stress**. Quantitative reverse transcription PCR (RT-qPCR) was used to validate the expression pattern of known miRNAs showing highly response to salt stress (Log2FC > 0.5). Gma-miR1515a was used as an internal control. The results shown are the averages ± SD of three biological replicates. Error bars indicate the standard deviation. Student's *t*-tests were performed; statistically significant results are marked with “^*^” (*P* < 0.05) and “^***^” (*P* < 0.001).

To validate the expression of these putative salt-responsive miRNAs in the RAM of soybean, we performed qRT-PCR using the samples prepared for Solexa sequencing. As shown in Figure 4, the selected salt-responsive miRNAs showed similar expression trends to those detected by Solexa sequencing. For example, high-level induction of miR390e and miR172f/g in the stressed RAM was reliably observed. By contrast, miR399a/b, miR1512b, and miR156g were stably and dramatically down-regulated in the RAM of salt stress-treated seedlings (Figure 4). Our results indicate that these known salt-responsive miRNAs may modulate RAM activity and subsequent plastic development of soybean roots in response to salt stress.

### Identification and validation of the novel miRNAs in response to salt stress

To identify the novel miRNAs, they were searched against a transcriptome database. Based on published criteria for miRNA annotation (Meyers et al., 2008), the potential miRNA precursors were searched in the soybean genome and their hairpin structures were predicted using miRDeep2. The lengths of the novel miRNA precursors ranged from 109 to 114 nt. Based on the criteria, 25 novel mature miRNAs belonging to 22 miRNA families were identified (Table S2); among these, 5 mature novel miRNAs began with a 5′ uridine. The miRNA^*^ abundance is usually low, and some of them cannot be detected by small RNA deep sequencing. Fortunately, we obtained both miRNA and miRNA^*^ sequences from our two libraries, although the miRNA^*^ sequences were less abundant compared with the miRNA sequences (Table S2). These results validate the occurrence of the novel miRNAs in soybean.

**Figure 5:**
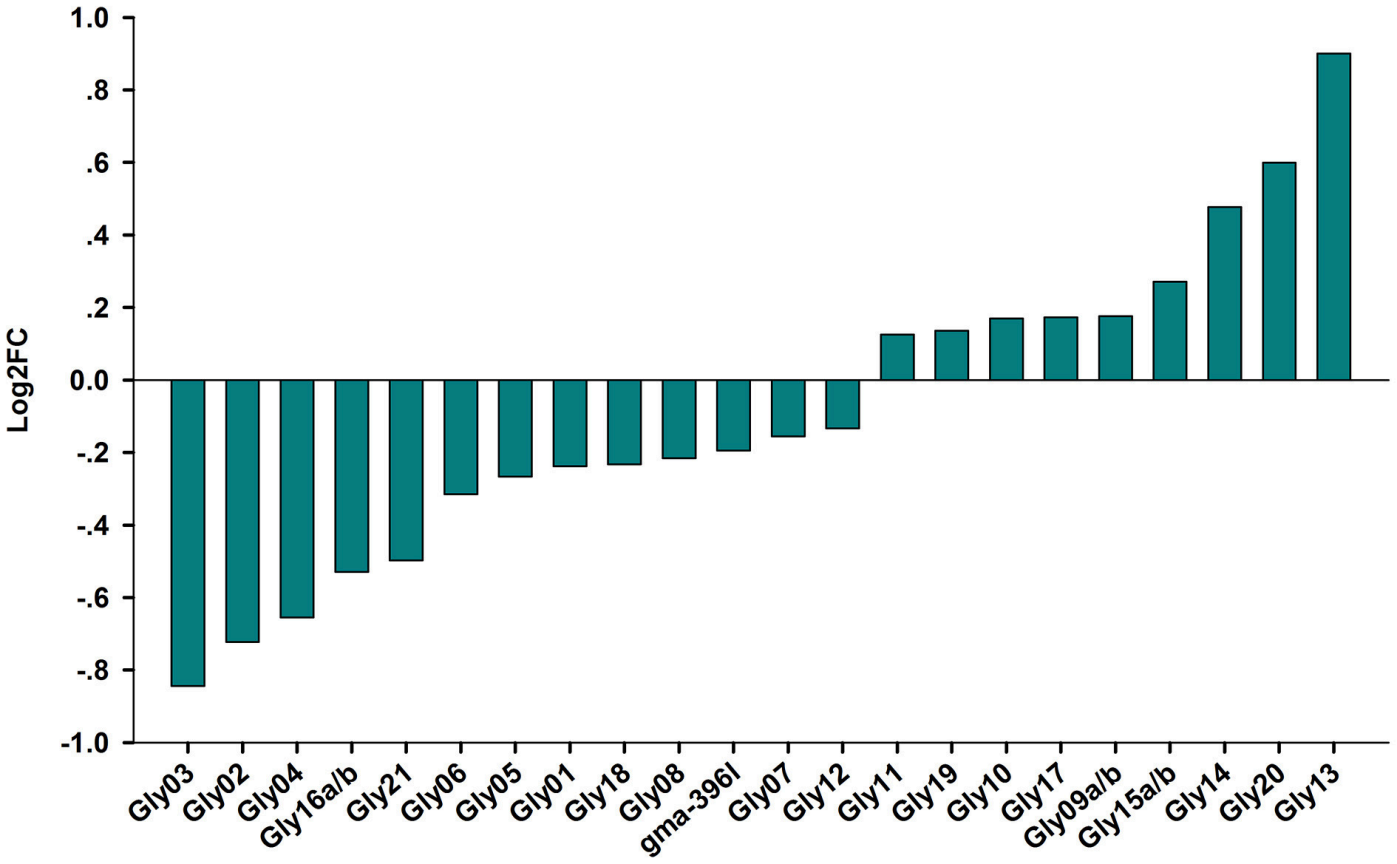
**Novel miRNAs expression level from Solexa sequencing**. Logarithmic fold change of novel miRNAs in response to salt stress in the sequencing results from CR and SR libraries.

**Figure 6:**
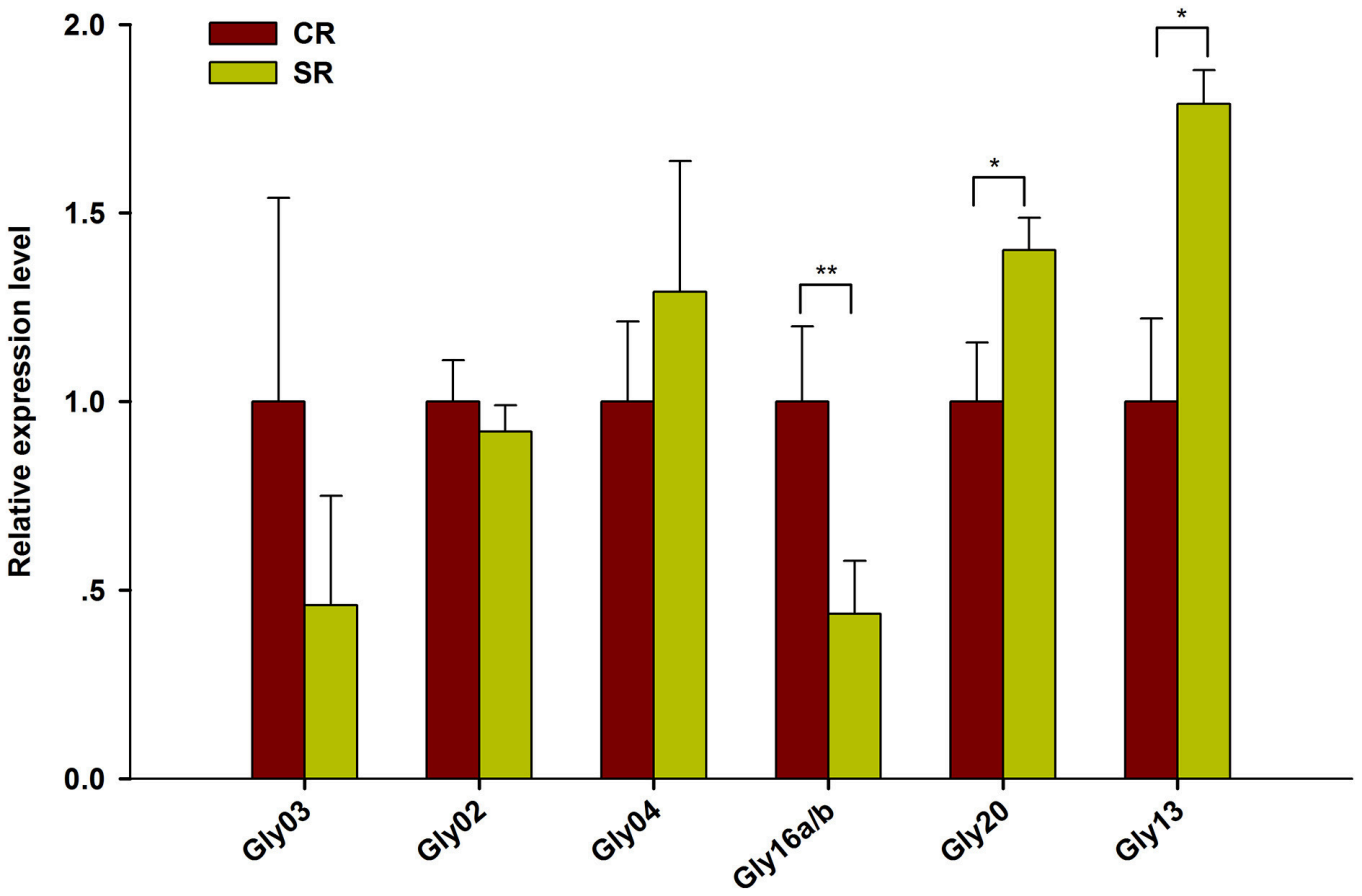
**Expression validation of salt stress responsive novel miRNAs using RT-qPCR**. Quantitative reverse transcription PCR (RT-qPCR) was used to analyze the expression pattern of novel miRNAs appeared to be highly response to salt stress (Log2FC > 0.5). Gma-miR1515a was used as an internal control. The results shown are the averages ± SD of three biological replicates. Error bars indicate the standard deviation. Student's *t*-tests were performed; statistically significant results are marked with “^*^” (*P* < 0.05), “^**^” (*P* < 0.01).

Among the novel miRNAs identified from the two libraries, two miRNAs were significantly up-regulated (log2FC > 0.5) and four miRNAs were dramatically down-regulated (log2FC < -0.5) under salt stress (Figure 5). Predicted hairpin structure of the six novel miRNA families precursors were shown in Figure S2. An analysis by qRT-PCR revealed that the expression of Gly03 and Gly16a/b was repressed by salt stress in the RAM of soybean plants treated with salt stress, while Gly20 was greatly up-regulated (Figure 6). This result is consistent with our sequence data with the exception of Gly04, whose expression was decreased under salt stress according to the sequence data but was slightly induced according to the qRT-PCR experiments (Figure 6).

### Many salt stress-responsive miRNAs are responsive to auxin

Due to the significant increase in auxin content in the stressed RAMs, we speculated that these salt stress-responsive miRNAs are also regulated by auxin. To test this possibility, we first analyzed the *cis* regulatory elements in the 2-kb promoter regions of 17 salt stress-responsive miRNAs. As shown in Table S3, the ARFAT *cis*-element, which is important for the auxin response, was present in the promoters of 13 miRNAs. Another two auxin-responsive *cis*-elements (NTBBF1ARROLB and CATATGGMSAUR) were found in the promoters of 16 miRNAs (Table S3). However, the *cis*-element CACGCAATGMGH3 was found only in the promoter of gma-miR172f (Table S3). Notably, the majority of these salt-responsive genes contained more than two auxin-responsive elements, suggesting that these miRNAs are regulated by auxin.

**Figure 7:**
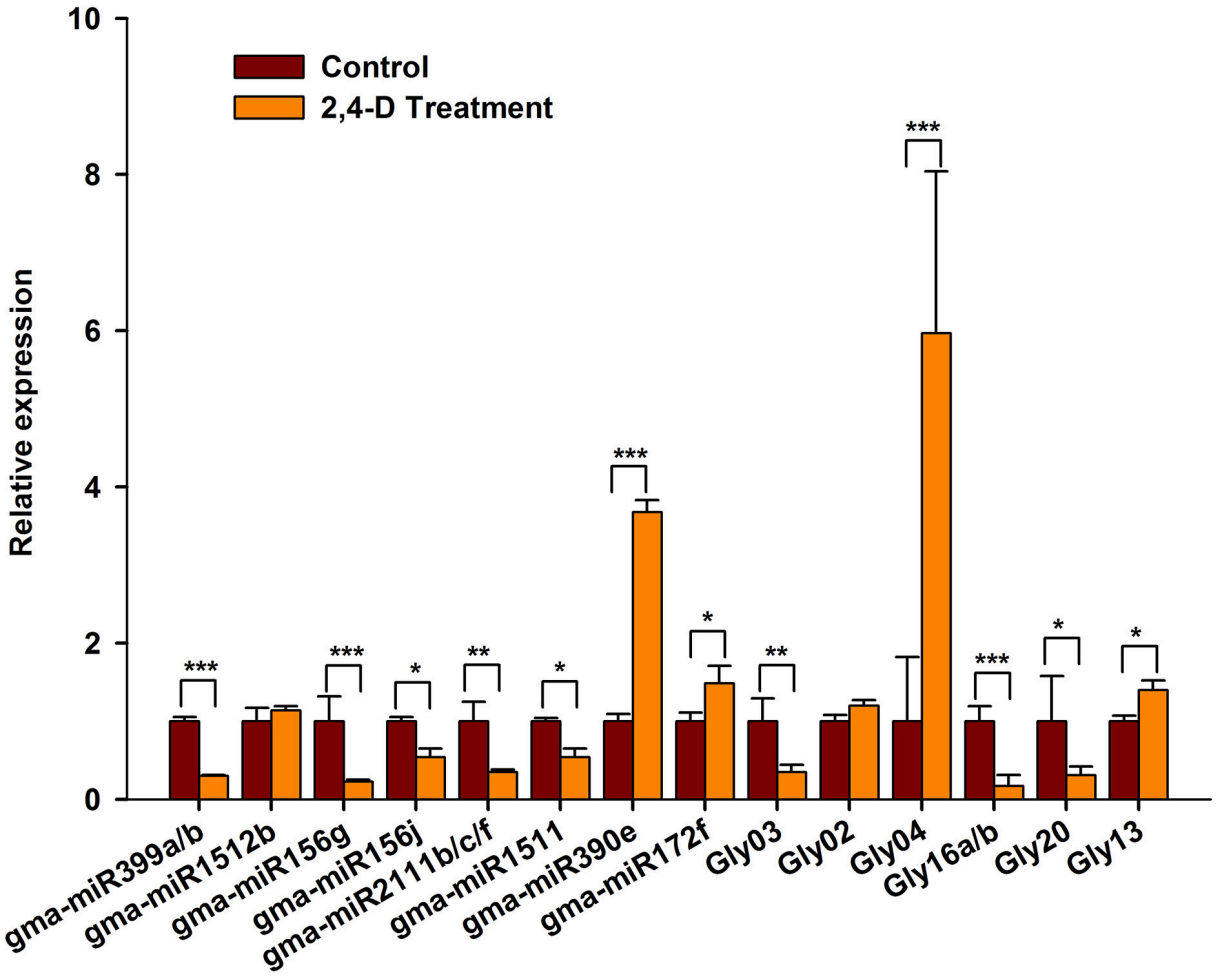
**The expression of salt stress responsive miRNAs was affected by exogenous auxin**. Five-day-old seedlings were transferred to liquid B5 medium supplemented with or without 1 μM 2,4-D. After 3 days, root tips were collected and used for RT-qPCR analysis. Gma-miR1515a was used as an internal control. The results shown are the averages ± SD of three biological replicates. Error bars indicate the standard deviation. Student's *t*-tests were performed; statistically significant results are marked with ^*^ (*P* < 0.05); ^**^ (*P* < 0.01); ^***^ (*P* < 0.001).

To verify our computational prediction, we analyzed the expression of the selected miRNAs in RAMs treated with exogenous auxin. As shown in Figure 7, the majority of the miRNAs were auxin-responsive. The expression of gma-miR399a/b, gma-miR156g/j, gma-miR2111b/c/f, gma-miR1511, Gly03, Gly16a/b Gly20 appeared to be significantly repressed by exogenous auxin, whereas that of gma-miR390e, Gly04, and Gly13 was induced by exogenous auxin (Figure 7). Intriguingly, the expression patterns of these miRNAs in response to auxin and salt stress were similar. However, some of the miRNAs showed different responses to exogenous auxin and salt stress. For example, gma-miR1512b and were repressed by salt stress, but they were unaffected by exogenous auxin (Figure 7). Gly20 was repressed by auxin but induced by salt stress. Taken together, our data suggest that these miRNAs act downstream of the auxin signaling pathway to modulate RAM activity in soybean under conditions of salt stress.

### Target prediction for the differentially expressed miRNAs

To further elucidate the possible mechanistic pathways or biological processes mediated by these miRNAs, we performed a bioinformatic analysis using TargetFinder to predict candidate targets for the known and novel miRNAs. According to the criteria described in the Section Materials and Methods, 80 putative target genes were predicted for the 13 differentially expressed known and novel miRNA families in the soybean RAM (Table S4). Most of the known and newly identified salt-responsive miRNAs were predicted to target classical transcription factors such as Squamosa promoter-binding-like protein for gma-miR156 and APETALA2, respectively (Table S4). In addition, the predicted targets included the genes encoding a leucine-rich repeat (LRR) receptor-like protein kinase, E3 ubiquitin ligase, F-box/kelch-repeat protein, the mitochondrial carrier protein PET8, and protein phosphatase 2C, suggesting that the miRNA-mediated regulation of the RAM under salt stress occurs at multiple levels in different locations.

Notably, we found that several known soybean miRNAs may target a greater number of genes than their homologs in *Arabidopsis*. In *Arabidopsis*, miR399 directly targets the protein PHO2 (UBC), which is an ubiquitin E2-conjugating enzyme (Bari et al., 2006). However, in addition to *Glyma.13G239100* (*PHO2*) and *Glyma.10G036800* (*GmPT5*), which have been validated as the targets of gma-miR399 (Xu et al., 2013), *Glyma.14G188000* and *Glyma.15G074200* annotated as Inorganic phosphate transporter and Ubiquitin-conjugatine enzyme E2 respectively, were also predicted to be the target genes of gma-miR399. In soybean, *Glyma.08G359400* and *Glyma18G177400* encoding multicopper oxidases and *Glyma03G021900* encoding a growth-regulating factor were also predicted to be putative targets of gma-miR399a/b (Table S4). We have detected the expression of these predicted target genes of gma-miR399a/b, as shown in Figure S3, *Glyma.03G021900, Glyma.08G359400, Glyma.14G188000*, and *Glyma.15G074200* were significantly induced by salt stress. Another known miRNA, gma-miR390e, was also predicted to target two additional genes (*Glyma.02G281100* and *Glyma.14G033500* encoding LRR receptor-like kinases in addition to *TAS3* (*Glyma.15G137400*) in soybean. Furthermore, we found that several novel miRNAs target genes that are involved in various biological processes. For example, Gly13 was predicted to target seven genes, including those encoding cysteine synthase A, ubiquitin-like protease protein 2, and a class I glutamine amidotransferase. Our results suggest that these soybean miRNAs modulate RAM activity by coordinating complicated regulatory networks.

### Gma-miR399a modulates plastic root development in soybean under salt stress

**Figure 8:**
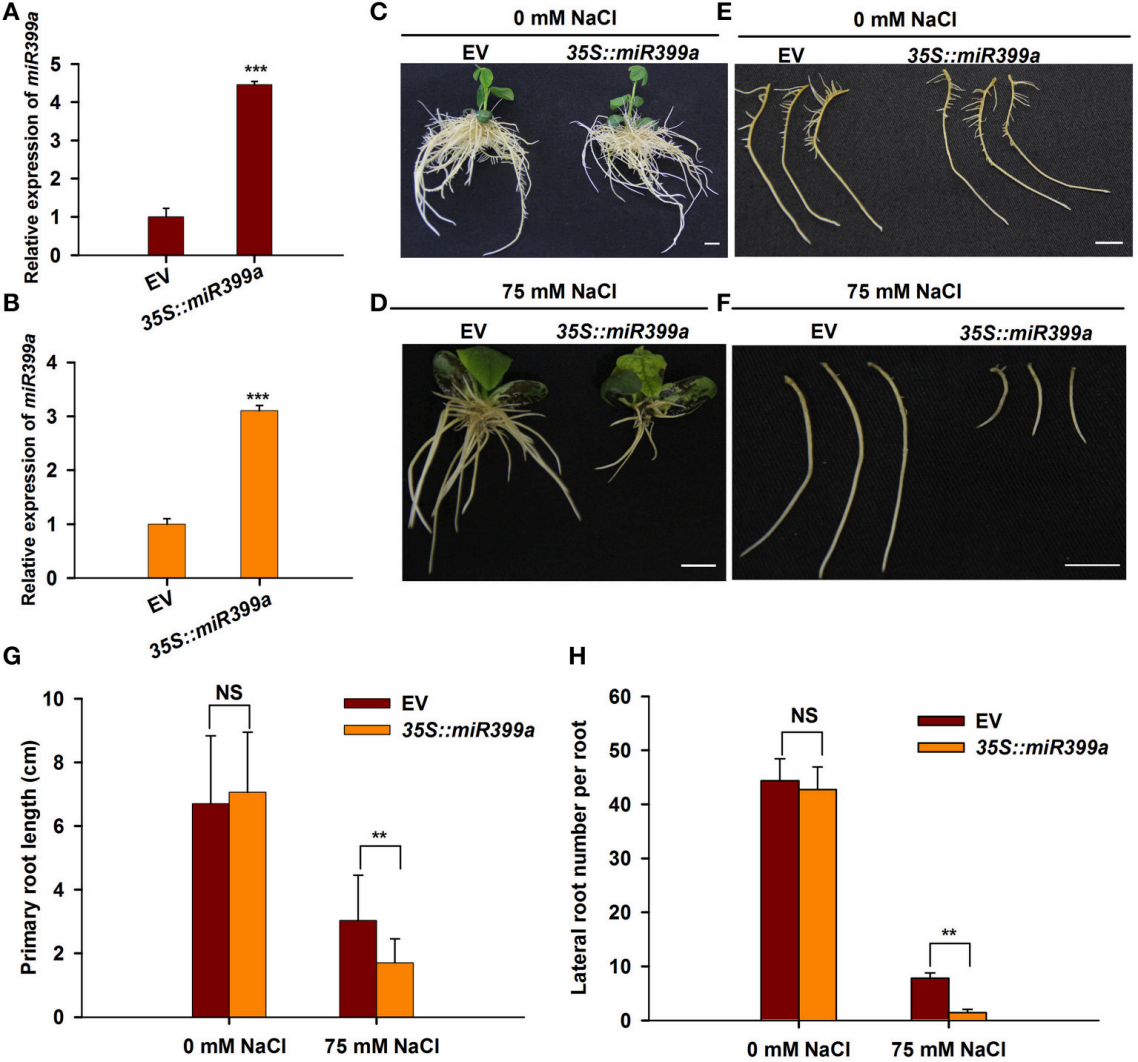
**Effects of overexpression gma-miR399a on root system architecture under salt stress**. **(A,B)** qRT-PCR analysis of miR399a relative expression in transgenic root expressing empty vector (pGWB2) and *35S::miR399a* under 0 and 75 mM NaCl treatment, respectively. **(C,D)** The phenotype of whole composite plants having empty vector or *35S::miR399a* in the rooting medium containing 0 or 75 mM NaCl. Pictures were taken at 12 days after transplantation. **(E,F)** The representative roots of empty vector or *35S::miR399a* were taken photos. Scale bar, 1 cm. The primary root length **(G)** and the lateral root number per hairy root **(H)** expressing empty vector or *35S::miR399a* were counted. The results given are averages ± SD of 3 biological replicates. Error bars indicate the standard deviation. Student's *t*-tests were performed; statistically significant results are marked with “^**^” (*P* < 0.01); and “^***^” (*P* < 0.001), “NS” means no significant difference.

It has been shown that miR399 family miRNAs mediate phosphate uptake in various plant species (Bari et al., 2006; Wang et al., 2009; Hackenberg et al., 2013). The fact that miR399a/b was highly responsive to salt stress prompted us to investigate whether miR399 also mediates root developmental plasticity in response to salt stress in soybean. To this end, we generated transgenic roots overexpressing gma-miR399a under the control of the CaMV35S promoter using a hairy root transformation system. A phenotypic analysis revealed that the transgenic roots overexpressing *35S::miR399a*, including primary and lateral roots, were comparable to those from plants carrying the empty vector control when grown under normal conditions (Figures 8C,E); in contrast, the roots from plants overexpressing *35S::miR399a* exhibited increased sensitivity to salt stress when treated with 75 mM NaCl (Figures 8D,F). Primary root growth in the *35S::miR399a*-overexpressing roots was reduced by 40%, and the lateral root number per hairy root was decreased by up to 70% under salt stress conditions (Figures 8A,B). These results suggest a crucial role for gma-miR399 in root developmental plasticity in soybean in response to salt stress.

## Discussion

Plant roots grow post-embryonically, and root growth and the root system architecture in plants are prominently controlled by environmental cues. Therefore, one of the mysteries in plant biology is the regulation of root development with a focus on RAM maintenance and remodeling of the RAM in response to various stresses. Using *Arabidopsis* as a model plant, several regulators of RAM activity, including PLT1/2, SHR, and SCR, have been identified (Helariutta et al., 2000; Aida et al., 2004). However, the complete regulatory network in the RAM is unclear. A more important question is how plant RAMs integrate developmental and environmental signals to reprogram RAM cell fate decisions in crop plants. In the current study, we demonstrated high plasticity of the miRNAome in the RAM of soybean plants in response to salt stress, and we revealed a complex regulatory network mediated by miRNAs that enables the soybean RAM to cope with salt stress.

There have been a couple of previous attempts to explore the regulatory networks that modulate root responses to salt stress, including a microarray analysis of different cell layers in *Arabidopsis* and a global analysis of the miRNAs in *M. truncatula* root tips under salt stress (Dinneny et al., 2008; Formey et al., 2014). The results of these studies suggested that the transcriptome and miRNAome of plant root tips are sensitive to salt stress, and that the plasticity of the transcriptome and miRNAome shows cell/tissue specificity, as well as species specificity. Here, we present important evidence to support the above hypothesis. We found that the soybean RAM and root system architecture exhibit great plasticity in response to moderate salt stress, and that morphological remodeling of the RAM is correlated with global reprogramming of the miRNAome. We confirmed that 19 miRNAs, including 11 known and 6 novel miRNAs, are responsive to salt stress in the soybean RAM. Among the known miRNAs, most (gma-miR399a/b, gma-miR156g, gma-miR156j, gma-miR2111b/c/f, gma-miR1511, gma-miR390e, and gma-miR172f) have been shown to be involved in root responses to salt stress in plants, except for gma-miR1512b, which is legume-specific (Li et al., 2010). These data demonstrate the important role of the miRNA-mediated regulatory network in the response of the soybean RAM to salt stress.

Plants constantly encounter various adverse soil stresses, and they have evolved dedicated regulatory networks that are responsible for adaptations at the cell, tissue, organ, and species levels. Our current results indicate that a given miRNA family or members of a family may be involved in diverse biological processes in different plant species. For example, miR2111 is a well-known miRNA that mediates plant responses to phosphate deprivation in Arabidopsis (Hsieh et al., 2009; Kuo and Chiou, 2011). However, our data show that this miRNA is responsive to salt stress in the soybean RAM (Figures 3, 4). Furthermore, we found that the same miRNA family may behave differently in mediating plant responses to salt stress. For example, mtr-miR390 was repressed by salt stress in *Medicago* root tips (Lelandais-Briere et al., 2009), however, glyma-miR390 was induced by salt stress in the soybean RAM (Figures 3, 4). In the case of miR399, these miRNAs may be involved in the general regulation of plant responses to environmental stresses, including phosphate starvation and salt stress. However, miR399 also shows a variable expression pattern in response to salt stress in different plants; it was shown to be induced by salt stress in *Populus tremula* (Jia et al., 2009), but repressed by salt in both *Medicago* and soybean. A functional analysis of glyma-miR399a further confirmed that miR399 is required for RAM and root development under salt stress conditions (Figure 8). These results demonstrate that the function of miR399 in the response to salt stress is conserved in legumes, and that functional divergence of these miRNAs may occur in different plant species. Although, legumes share a more conserved regulatory mechanism for coping with salt stress, our study identified miR1512 as another miRNA that specifically regulates the soybean RAM response to salt stress. Previous data have shown that miR1512 is a legume-specific miRNA, but no reports have shown that miR1512 is involved in the salt response of legumes, including *Medicago* root tips. We found that gma-miR1512b was significantly down-regulated upon salt stress treatment in the root tip of soybean, suggesting that gma-miR1512 plays an important and specific role in plastic root development in soybean under conditions of salt stress. Additional studies of gma-miR1512 will further our understanding of how the soybean RAM responds to salt stress.

One efficient way to elucidate miRNA-mediated biological processes and plant stress responses is to find their target genes. Based on computational predictions, we identified putative target genes for all salt-responsive miRNAs in the soybean RAM. As expected, some salt-responsive miRNAs identified in this study, including gma-miR156 and gma-miR172, target the same family of genes encoding various proteins (e.g., AP2 transcription factors; Table S4). Unexpectedly, we found that some miRNAs may target more diverse gene families in soybean. For example, miR390 directly targets the non-coding TAS3 precursor RNA to trigger the biogenesis of tasiRNAs-ARF, which cleave the transcripts of ARF2, ARF3, and ARF4, thereby regulating lateral root development (Chen and Xiong, 2010; Marin et al., 2010). In soybean, we found that miR390 has additional putative target genes with diverse functions, except the *Arabidopsis* TAS homolog. One target gene encodes a protein with strong similarity to AtBAM3, a receptor kinase-like protein that regulates shoot and floral meristem development (Depuydt et al., 2013). Taking into account the role of AtBAM3 in meristems, it is likely that gma-miR390 modulates RAM activity and root developmental plasticity through an *AtBAM3* homolog in soybean. Gma-miR399 also has additional putative target genes with different biochemical features beyond the well-known target gene *PHO2*, which is involved in plant responses to phosphate starvation. Based on our results, *GmPHO2* has no significantly change in response to salt stress but another homologous gene *Glyma.15G074200* was obviously induced under salt stress. In *Arabidopsis*, the homologous gene of *Glyma.03G021900* function as GROWTH-REGULATING FACTOR 9, regulates the plant growth and development process (Horiguchi et al., 2005). *Glyma.08G359400* was annotated as a multicopper oxidase which might play roles in plants development to response abiotic stress and phosphate sensing in the root tip (Svistoonoff et al., 2007; Desnos, 2008). We found both of these genes were induced by salt stress in the root tip of soybean (Figure S3). These data suggest that gma-miR399a/b may participate in the plastic development of RAM under salt stress through target one or more genes. These findings suggest that miRNAs modulate plant growth and stress responses via a complicated network in soybean that includes the negative regulation of functionally diverse target genes.

Despite the great importance of miRNA-mediated regulatory networks in plant growth and adaptation, the upstream regulators of these key miRNAs remain largely unknown. In the present study, we found that salt stress induced auxin accumulation in the soybean RAM (Figure 1D). Intriguingly, we produced two pieces of evidence showing that auxin and the auxin signaling pathway act upstream of these miRNAs in response to salt stress. First, most of the salt-responsive miRNAs contain at least one auxin-responsive *cis*-element (Table S3). Second, we experimentally confirmed that these miRNAs are indeed responsive to exogenous auxin, and that the expression patterns of the miRNAs were similar to those under salt stress (Figure 7). It is well known that auxin plays an essential role in the regulation of the RAM and root plastic development (Aloni et al., 2006). To date, the auxin-PLT pathway is the only well-characterized pathway for RAM regulation in *Arabidopsis* (Aloni et al., 2006). To date, the auxin-PLT pathway is the only well-characterized pathway for RAM regulation in *Arabidopsis* (Aida et al., 2004). Except for a few miRNAs (miR160, miR167, and miR393) that directly target components of the auxin signaling pathway or auxin-responsive genes, our data establish a new link between auxin signaling and a broad spectrum of biochemical and cellular processes via various miRNAs.

The maintenance of RAM activity is essential for indeterminate root growth in plants, and the reprogramming of RAM activity is crucial for ensuring the correct root system architecture and survival of plants in a constantly changing environment. The current report reveals the critical role of miRNAome plasticity in the soybean RAM in response to salt stress. We propose that the salt-induced activation of auxin signaling is an important upstream event that mediates RAM remodeling through targeting by salt-responsive miRNAs in soybean.

## Author contributions

YW and XL conceived the study and designed the experiments. ZS, YW, FM, YT, LC, SZ, and QJ performed the experiments. YW, FM, YT, LC, SZ, and QJ collected the samples for high-throughput sequencing. ZS, YW, and LC performed the expression validation of the miRNAs and phenotypic analyses of the transgenic roots. ZS, YW, and FM analyzed the experimental data. ZS, YW, and XL wrote the article.

### Conflict of interest statement

The authors declare that the research was conducted in the absence of any commercial or financial relationships that could be construed as a potential conflict of interest.

## Supplementary material

Figure S1 **The effect of salt stress to root apical meristem of soybean**. Longitudinal paraffin section of root tips (0.5 cm) collected from medium containing 0 mM NaCl **(left)** and 75 mM NaCl **(right)**. Blue arrow points the position dividing root elongation zone and meristem zone. Bar = 100 μm.

Figure S2 **Predicted RNA hairpin structures of novel miRNA precursors**. Precursor structures of salt stress responsive novel miRNAs were predicted by miRDeep2. Mature miRNAs are highlighted in red and miRNA^*^ in purple, and yellow for loop structure.

Figure S3 **Expression analysis of predicted target genes of gma-miR399a/b in response to salt stress**.

Table S1 **List of known miRNAs identified from root tips of ***Glycine max*** with or without salt stress**.

Table S2 **List of novel miRNAs identified from root tips of ***Glycine max*** with or without salt stress**.

Table S3 **Prediction of ***cis***-element in the promoters of salt stress responsive miRNAs**.

Table S4 **Putative target genes of known salt stress responsive miRNAs**.

Table S5 **The list of primers used in experiment**.

## References

[B1] Aghaei K. Ehsanpour A. A. Shah A. H. Komatsu S. (2009). Proteome analysisof soybean hypocotyl and root under salt stress. Amino. Acids 36, 91–98. 10.1007/s00726-008-0036-7 18264660

[B2] Aida M. Beis D. Heidstra R. Willemsen V. Blilou I. Galinha C.. (2004). The PLETHORA genes mediate patterning of the Arabidopsis root stem cell niche. Cell 119, 109–120. 10.1016/j.cell.2004.09.018 15454085

[B3] Aloni R. Aloni E. Langhans M. Ullrich C. I. (2006). Role of cytokinin and auxin in shaping root architecture: regulating vascular differentiation, lateral root initiation, root apical dominance and root gravitropism. Ann. Bot. 97, 883–893. 10.1093/aob/mcl027 16473866 PMC2803412

[B4] Bari R. Datt Pant B. Stitt M. Scheible W. R. (2006). PHO2, microRNA399, and PHR1 define a phosphate-signaling pathway in plants. Plant Physiol. 141, 988–999. 10.1104/pp.106.079707 16679424 PMC1489890

[B5] Bartel D. P. (2004). microRNAs: genomics, biogenesis, mechanism, and function. Cell 116, 281–297. 10.1016/S0092-8674(04)00045-5 14744438

[B6] Bazin J. Khan G. A. Combier J. P. Bustos-Sanmamed P. Debernardi J. M. Rodriguez R.. (2013). miR396 affects mycorrhization and root meristem activity in the legume *Medicago truncatula*. Plant J. 74, 920–934. 10.1111/tpj.12178 23566016

[B7] Burgos N. R. Talbert R. E. Kim K. S. Kuk Y. I. (2004). Growth inhibition and root ultrastructure of cucumber seedlings exposed to allelochemicals from rye (*Secale cereale*). J. Chem. Ecol. 30, 671–689. 10.1023/B:JOEC.0000018637.94002.ba 15139316

[B8] Bustos-Sanmamed P. Mao G. Deng Y. Elouet M. Khan G. A. Bazin J. (2013). Overexpression of miR160 affects root growth and nitrogen-fixing nodule number in *Medicago truncatula*. Funct. Plant Biol. 40, 1208 10.1071/FP13123 32481189

[B9] Chen C. Ridzon D. A. Broomer A. J. Zhou Z. Lee D. H. Nguyen J. T.. (2005). Real-time quantification of microRNAs by stem-loop RT-PCR. Nucleic Acids Res. 33, e179. 10.1093/nar/gni178 16314309 PMC1292995

[B10] Chen H. Xiong L. (2010). The bifunctional abiotic stress signalling regulator and endogenous RNA silencing suppressor FIERY1 is required for lateral root formation. Plant Cell Environ. 33, 2180–2190. 10.1111/j.1365-3040.2010.02218.x 20807376

[B11] Cui L. G. Shan J. X. Shi M. Gao J. P. Lin H. X. (2014). The miR156-SPL9-DFR pathway coordinates the relationship between development and abiotic stress tolerance in plants. Plant J. 80, 1108–1117. 10.1111/tpj.12712 25345491

[B12] D'alessandro S. Golin S. Hardtke C. S. Lo Schiavo F. Zottini M. (2015). The co-chaperone p23 controls root development through the modulation of auxin distribution in the Arabidopsis root meristem. J. Exp. Bot. 66, 5113–5122. 10.1093/jxb/erv330 26163704 PMC4513928

[B13] Depuydt S. Rodriguez-Villalon A. Santuari L. Wyser-Rmili C. Ragni L. Hardtke C. S. (2013). Suppression of *Arabidopsis* protophloem differentiation and root meristem growth by CLE45 requires the receptor-like kinase BAM3. Proc. Natl. Acad. Sci. U.S.A. 110, 7074–7079. 10.1073/pnas.1222314110 23569225 PMC3637694

[B14] Desnos T. (2008). Root branching responses to phosphate and nitrate. Curr. Opin. Plant Biol. 11, 82–87. 10.1016/j.pbi.2007.10.003 18024148

[B15] De Tullio M. C. Jiang K. Feldman L. J. (2010). Redox regulation of root apical meristem organization: connecting root development to its environment. Plant Physiol. Biochem. 48, 328–336. 10.1016/j.plaphy.2009.11.005 20031434

[B16] Ding D. Zhang L. Wang H. Liu Z. Zhang Z. Zheng Y. (2009). Differential expression of miRNAs in response to salt stress in maize roots. Ann. Bot. 103, 29–38. 10.1093/aob/mcn205 18952624 PMC2707283

[B17] Ding Z. Friml J. (2010). Auxin regulates distal stem cell differentiation in Arabidopsis roots. Proc. Natl. Acad. Sci. U.S.A. 107, 12046–12051. 10.1073/pnas.1000672107 20543136 PMC2900669

[B18] Dinneny J. R. Benfey P. N. (2008). Plant stem cell niches: standing the test of time. Cell 132, 553–557. 10.1016/j.cell.2008.02.001 18295573

[B19] Dinneny J. R. Long T. A. Wang J. Y. Jung J. W. Mace D. Pointer S.. (2008). Cell identity mediates the response of *Arabidopsis* roots to abiotic stress. Science 320, 942–945. 10.1126/science.1153795 18436742

[B20] Ferguson B. J. Indrasumunar A. Hayashi S. Lin M. H. Lin Y. H. Reid D. E.. (2010). Molecular analysis of legume nodule development and autoregulation. J. Integr. Plant Biol. 52, 61–76. 10.1111/j.1744-7909.2010.00899.x 20074141

[B21] Fernández-Marcos M. Sanz L. Lewis D. R. Muday G. K. Lorenzo O. (2011). Nitric oxide causes root apical meristem defects and growth inhibition while reducing PIN-FORMED 1 (PIN1)-dependent acropetal auxin transport. Proc. Natl. Acad. Sci. U.S.A. 108, 18506–18511. 10.1073/pnas.1108644108 22021439 PMC3215072

[B22] Formey D. Sallet E. Lelandais-Brière C. Ben C. Bustos-Sanmamed P. Niebel A.. (2014). The small RNA diversity from *Medicago truncatula* roots under biotic interactions evidences the environmental plasticity of the miRNAome. Genome Biol. 15:457. 10.1186/s13059-014-0457-4 25248950 PMC4212123

[B23] Fu J. Chu J. Sun X. Wang J. Yan C. (2012). Simple, rapid, and simultaneous assay of multiple carboxyl containing phytohormones in wounded tomatoes by UPLC-MS/MS using single SPE purification and isotope dilution. Anal. Sci. 28, 1081–1087. 10.2116/analsci.28.1081 23149609

[B24] Ge Y. Li Y. Zhu Y. M. Bai X. Lv D. K. Guo D.. (2010). Global transcriptome profiling of wild soybean (*Glycine soja*) roots under NaHCO_3_treatment. BMC Plant Biol. 10:153. 10.1186/1471-2229-10-153 20653984 PMC3017823

[B25] Grunewald W. Smet I. D. Lewis D. R. Löfke C. Jansen L. Goeminne G.. (2012). Transcription factor WRKY23 assists auxin distribution patterns during Arabidopsis root development through local control on flavonol biosynthesis. Proc. Natl. Acad. Sci. U.S.A. 109, 1554–1559. 10.1073/pnas.1121134109 22307611 PMC3277162

[B26] Guan R. Qu Y. Guo Y. Yu L. Liu Y. Jiang J.. (2014). Salinity tolerance in soybean is modulated by natural variation in GmSALT3. Plant J. 80, 937–950. 10.1111/tpj.12695 25292417

[B27] Hackenberg M. Shi B.-J. Gustafson P. Langridge P. (2013). Characterization of phosphorus-regulated miR399 and miR827 and their isomirs in barley under phosphorus-sufficient and phosphorus-deficient conditions. BMC Plant Biol. 13:214. 10.1186/1471-2229-13-214 24330740 PMC3878733

[B28] Helariutta Y. Fukaki H. Wysocka-Diller J. Nakajima K. Jung J. Sena G.. (2000). The shoot-root gene controls radial patterning of the *Arabidopsis* root through radial signaling. Cell 101, 555–567. 10.1016/S0092-8674(00)80865-X 10850497

[B29] Horiguchi G. Kim G. Tsukaya H. (2005). The transcription factor AtGRF5 and the transcription coactivator AN3 regulate cell proliferation in leaf primordia of *Arabidopsis thaliana*. Plant J. 43, 68–78. 10.1111/j.1365-313X.2005.02429.x 15960617

[B30] Hsieh L. C. Lin S. I. Shih A. C. Chen J. W. Lin W. Y. Tseng C. Y.. (2009). Uncovering small RNA-mediated responses to phosphate deficiency in *Arabidopsis* by deep sequencing. Plant Physiol. 151, 2120–2132. 10.1104/pp.109.147280 19854858 PMC2785986

[B31] Im J. H. Lee H. Kim J. Kim H. B. An C. S. (2012). Soybean MAPK, GMK1 is dually regulated by phosphatidic acid and hydrogen peroxide and translocated to nucleus during salt stress. Mol. Cells 34, 271–278. 10.1007/s10059-012-0092-4 22886763 PMC3887844

[B32] Ji H. Liu L. Li K. Xie Q. Wang Z. Zhao X.. (2014). PEG-mediated osmotic stress induces premature differentiation of the root apical meristem and outgrowth of lateral roots in wheat. J. Exp. Bot. 65, 4863–4872. 10.1093/jxb/eru255 24935621 PMC4144773

[B33] Jia X. Wang W. X. Ren L. Chen Q. J. Mendu V. Willcut B.. (2009). Differential and dynamic regulation of miR398 in response to ABA and salt stress in *Populus tremula* and *Arabidopsis thaliana*. Plant Mol. Biol. 71, 51–59. 10.1007/s11103-009-9508-8 19533381

[B34] Jian B. Hou W. Wu C. Liu B. Liu W. Song S.. (2009). *Agrobacterium rhizogenes*-mediated transformation of Superroot-derived *Lotus corniculatus* plants: a valuable tool for functional genomics. BMC Plant Biol. 9:78. 10.1186/1471-2229-9-78 19555486 PMC2708162

[B35] Jiang K. Feldman L. (2002). Root meristem establishment and maintenance: the role of auxin. J. Plant Growth Regul. 21, 432–440. 10.1007/s00344-002-0037-9

[B36] Kereszt A. Li D. Indrasumunar A. Nguyen C. D. T. Nontachaiyapoom S. Kinkema M.. (2007). *Agrobacterium rhizogenes*-mediated transformation of soybean to study root biology. Nat. Protoc. 2, 948–952. 10.1038/nprot.2007.141 17446894

[B37] Kerk N. M. Jiang K. Feldman L. J. (2000). Auxin metabolism in the root apical meristem. Plant Physiol. 122, 925–932. 10.1104/pp.122.3.925 10712557 PMC58929

[B38] Khraiwesh B. Zhu J. K. Zhu J. (2012). Role of miRNAs and siRNAs in biotic and abiotic stress responses of plants. Biochim. Biophys. Acta 1819, 137–148. 10.1016/j.bbagrm.2011.05.001 21605713 PMC3175014

[B39] Kulcheski F. R. Marcelino-Guimaraes F. C. Nepomuceno A. L. Abdelnoor R. V. Margis R. (2010). The use of microRNAs as reference genes for quantitative polymerase chain reaction in soybean. Anal. Biochem. 406, 185–192. 10.1016/j.ab.2010.07.020 20670612

[B40] Kuo H. F. Chiou T. J. (2011). The role of microRNAs in phosphorus deficiency signaling. Plant Physiol. 156, 1016–1024. 10.1104/pp.111.175265 21562333 PMC3135939

[B41] Lavenus J. Goh T. Roberts I. Guyomarc'h S. Lucas M. De Smet I.. (2013). Lateral root development in *Arabidopsis*: fifty shades of auxin. Trends Plant Sci. 18, 450–458. 10.1016/j.tplants.2013.04.006 23701908

[B42] Lelandais-Briere C. Naya L. Sallet E. Calenge F. Frugier F. Hartmann C.. (2009). Genome-wide *Medicago truncatula* small RNA analysis revealed novel microRNAs and isoforms differentially regulated in roots and nodules. Plant Cell 21, 2780–2796. 10.1105/tpc.109.068130 19767456 PMC2768930

[B43] Li H. Deng Y. Wu T. Subramanian S. Yu O. (2010). Misexpression of miR482, miR1512, and miR1515 increases soybean nodulation. Plant Physiol. 153, 1759–1770. 10.1104/pp.110.156950 20508137 PMC2923892

[B44] Li W. Wang T. Zhang Y. Li Y. (2016). Overexpression of soybean miR172c confers water deficit and salt tolerance but ABA sensitivity in transgenic *Arabidopsis thaliana*. J. Exp. Bot. 67, 175–194. 10.1093/jxb/erv450 26466661

[B45] Liu W. Li R. J. Han T. T. Cai W. Fu Z. W. Lu Y. T. (2015). Salt stress reduces root meristem size by nitric oxide-mediated modulation of auxin accumulation and signaling in *Arabidopsis*. Plant Physiol. 168, 343–356. 10.1104/pp.15.00030 25818700 PMC4424022

[B46] Liu Y. Lai N. Gao K. Chen F. Yuan L. Mi G. (2013). Ammonium inhibits primary root growth by reducing the length of meristem and elongation zone and decreasing elemental expansion rate in the root apex in *Arabidopsis thaliana*. PLoS ONE 8:e61031. 10.1371/journal.pone.0061031 23577185 PMC3620058

[B47] Lu Y. B. Yang L. T. Qi Y. P. Li Y. Li Z. Chen Y. B.. (2014). Identification of boron-deficiency-responsive microRNAs in *Citrus sinensis* roots by Illumina sequencing. BMC Plant Biol. 14:123. 10.1186/1471-2229-14-123 24885979 PMC4041134

[B48] Marchant A. Bhalerao R. Casimiro I. Eklöf J. Casero P. J. Bennett M.. (2002). AUX1 promotes lateral root formation by facilitating indole-3-acetic acid distribution between sink and source tissues in the *Arabidopsis* seedling. Plant Cell 14, 589–597. 10.1105/tpc.010354 11910006 PMC150581

[B49] Marin E. Jouannet V. Herz A. Lokerse A. S. Weijers D. Vaucheret H.. (2010). miR390, Arabidopsis TAS3 tasiRNAs, and their AUXIN RESPONSE FACTOR targets define an autoregulatory network quantitatively regulating lateral root growth. Plant Cell 22, 1104–1117. 10.1105/tpc.109.072553 20363771 PMC2879756

[B50] Meyers B. C. Axtell M. J. Bartel B. Bartel D. P. Baulcombe D. Bowman J. L.. (2008). Criteria for annotation of plant microRNAs. Plant Cell 20, 3186–3190. 10.1105/tpc.108.064311 19074682 PMC2630443

[B51] Perilli S. Di Mambro R. Sabatini S. (2012). Growth and development of the root apical meristem. Curr. Opin. Plant Biol. 15, 17–23. 10.1016/j.pbi.2011.10.006 22079783

[B52] Petricka J. J. Winter C. M. Benfey P. N. (2012). Control of *Arabidopsis* root development. Annu. Rev. Plant Biol. 63, 563–590. 10.1146/annurev-arplant-042811-105501 22404466 PMC3646660

[B53] Rao S. S. El-Habbak M. H. Havens W. M. Singh A. Zheng D. Vaughn L.. (2014). Overexpression of GmCaM4 in soybean enhances resistance to pathogens and tolerance to salt stress. Mol. Plant Pathol. 15, 145–160. 10.1111/mpp.12075 24118726 PMC6638926

[B54] Singh A. Singh S. Panigrahi K. C. Reski R. Sarkar A. K. (2014). Balanced activity of microRNA166/165 and its target transcripts from the class III homeodomain-leucine zipper family regulates root growth in *Arabidopsis thaliana*. Plant Cell Rep. 33, 945–953. 10.1007/s00299-014-1573-z 24504657

[B55] Sobhanian H. Razavizadeh R. Nanjo Y. Ehsanpour A. A. Jazii F. R. Motamed N.. (2010). Proteome analysis of soybean leaves, hypocotyls and roots under salt stress. Proteome Sci. 8:19. 10.1186/1477-5956-8-19 20350314 PMC2859372

[B56] Sunkar R. Li Y. F. Jagadeeswaran G. (2012). Functions of microRNAs in plant stress responses. Trends Plant Sci. 17, 196–203. 10.1016/j.tplants.2012.01.010 22365280

[B57] Svistoonoff S. Creff A. Reymond M. Sigoillot-Claude C. Ricaud L. Blanchet A.. (2007). Root tip contact with low phosphate media reprograms plant root architecture. Nat. Genet. 39, 792–796. 10.1038/ng2041 17496893

[B58] Turner M. Adhikari S. Subramanian S. (2013). Optimizing stem-loop qPCR assays through multiplexed cDNA synthesis of U6 and miRNAs. Plant Signal Behav. 8:e24918. 10.4161/psb.24918 23673353 PMC4010539

[B59] Ubeda-Tomas S. Bennett M. J. (2010). Plant development: size matters, and it's all down to hormones. Curr. Biol. 20, R511–R513. 10.1016/j.cub.2010.05.013 20620902

[B60] Wang Z. Hu H. Huang H. Duan K. Wu Z. Wu P. (2009). Regulation of OsSPX1 and OsSPX3 on expression of OsSPX domain genes and Pi-starvation signaling in rice. J. Integr. Plant Biol. 51, 663–674. 10.1111/j.1744-7909.2009.00834.x 19566645

[B61] West G. Inzé D. Beemster G. T. (2004). Cell cycle modulation in the response of the primary root of Arabidopsis to salt stress. Plant Physiol. 135, 1050–1058. 10.1104/pp.104.040022 15181207 PMC514139

[B62] Xie F. Stewart C. N. Jr. Taki F. A. He Q. Liu H. Zhang B. (2014). High-throughput deep sequencing shows that microRNAs play important roles in switchgrass responses to drought and salinity stress. Plant Biotechnol. J. 12, 354–366. 10.1111/pbi.12142 24283289

[B63] Xu F. Liu Q. Chen L. Kuang J. Walk T. Wang J.. (2013). Genome-wide identification of soybean microRNAs and their targets reveals their organ-specificity and responses to phosphate starvation. BMC Genomics 14:66. 10.1186/1471-2164-14-66 23368765 PMC3673897

[B64] Yasuta Y. Kokubun M. (2014). Salinity tolerance of super-nodulating soybean genotype En-b0-1. Plant Prod. Sci. 17, 32–40. 10.1626/pps.17.32

[B65] Zhang W. Swarup R. Bennett M. Schaller G. E. Kieber J. J. (2013). Cytokinin induces cell division in the quiescent center of the Arabidopsis root apical meristem. Curr. Biol. 23, 1979–1989. 10.1016/j.cub.2013.08.008 24120642

