# Supplementary figures and images for: Genome-Wide Small RNA Analysis of Soybean Reveals Auxin-Responsive microRNAs that are Differentially Expressed in Response to Salt Stress in Root Apex
Source: Front Plant Sci. 2016 Jan 18;6:1273. doi: 10.3389/fpls.2015.01273 (PMC4716665; doi:10.3389/fpls.2015.01273)

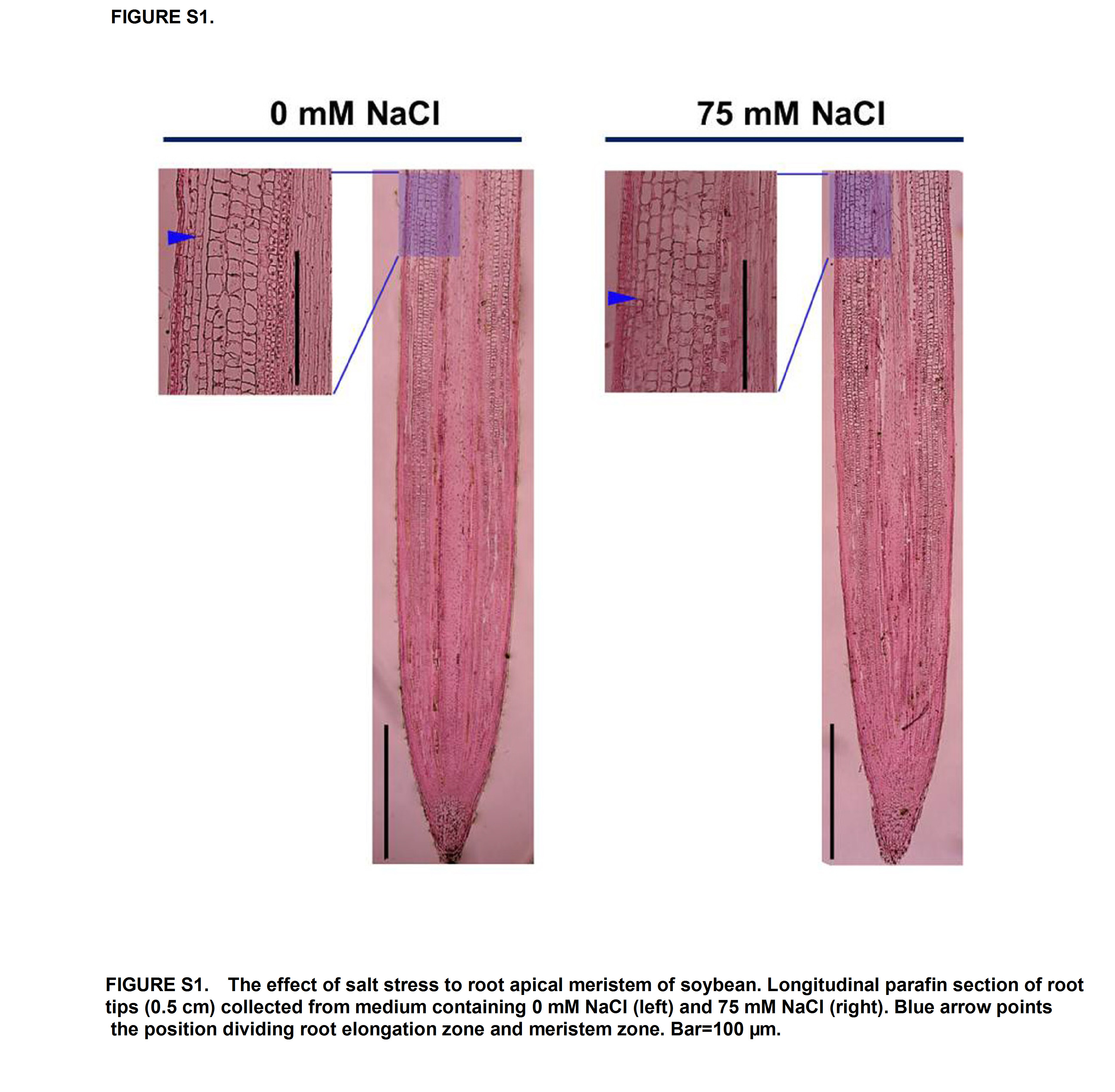

Supplement: Figure S1 — The effect of salt stress to root apical meristem of soybean. Longitudinal paraffin section of root tips (0.5 cm) collected from medium containing 0 mM NaCl (left) and 75 mM NaCl (right). Blue arrow points the position dividing root elongation zone and meristem zone. Bar = 100 μm. [file Image1.JPEG]

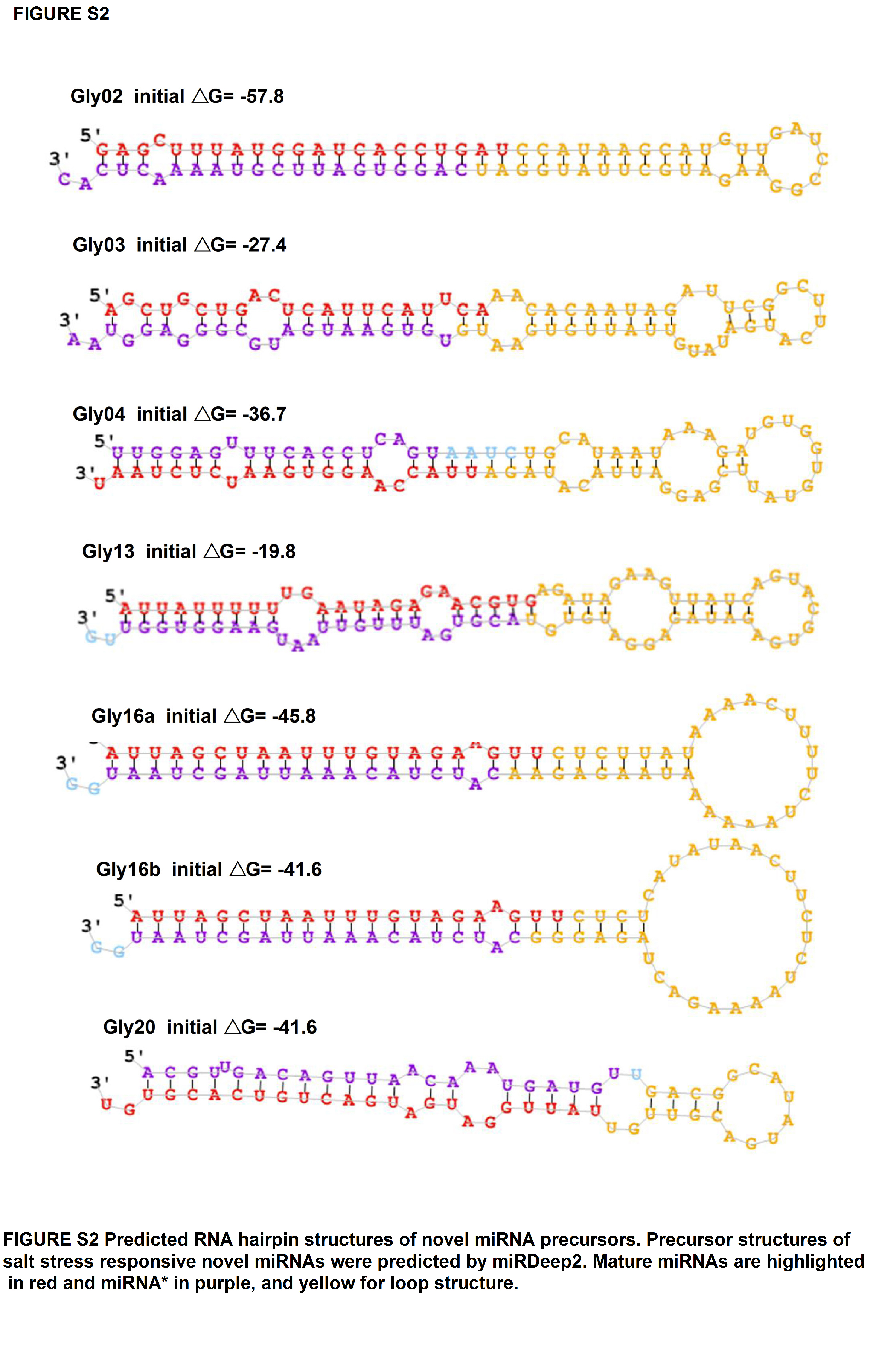

Supplement: Figure S2 — Predicted RNA hairpin structures of novel miRNA precursors. Precursor structures of salt stress responsive novel miRNAs were predicted by miRDeep2. Mature miRNAs are highlighted in red and miRNA* in purple, and yellow for loop structure. [file Image2.JPEG]

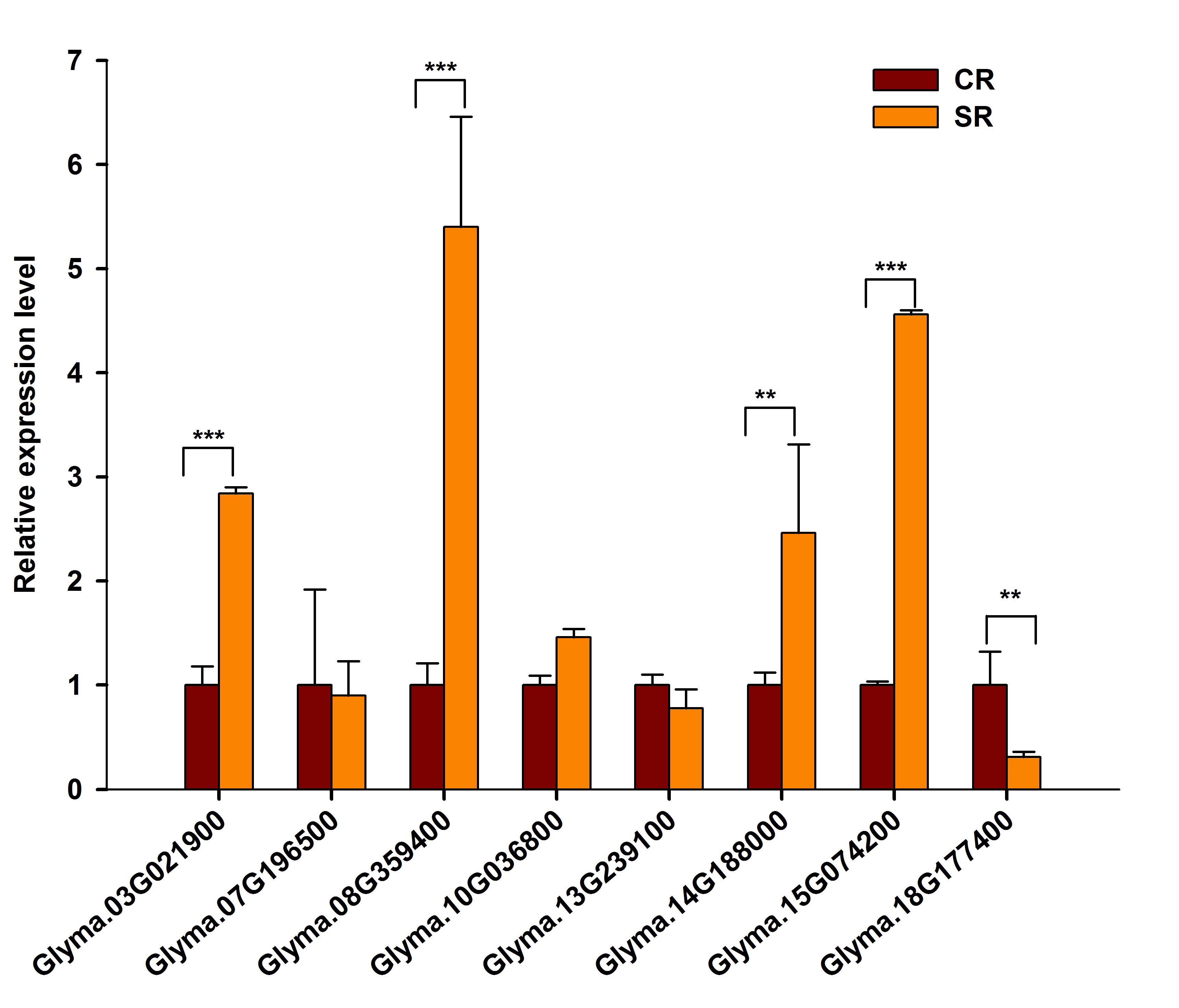

Supplement: Figure S3 — Expression analysis of predicted target genes of gma-miR399a/b in response to salt stress. [file Image3.JPEG]
